# Comprehensive analysis of the bacterial spectrum for enhanced clinical insight in microbial ureteral stent colonization, uncomplicated urinary tract infections and catheter-associated urinary tract infections: a principal component analysis-based literature review

**DOI:** 10.1007/s00345-024-05354-x

**Published:** 2024-12-12

**Authors:** Matilde Lepori, Olivier Braissant, Gernot Bonkat, Malte Rieken

**Affiliations:** 1 Department of Biomedical Engineering, University of Basel, Hegenheimermattweg 167B/C, 4123 Allschwil, Switzerland; 2 Alta Uro AG, Centralbahnplatz 6, 4051 Basel, Switzerland; 3 Faculty of Medicine, University of Basel, Basel, Switzerland

**Keywords:** Urinary tract infections, Bacteriuria, Ureteral catheterization, Ureteroscopy

## Abstract

**Purpose:**

Controversies exist regarding the prevailing spectrum of microorganisms in microbial ureteral stent colonization (MUSC) and their clinical significance. The aim of this comprehensive review is to determine the predominant microbial spectrum in patients with an indwelling ureteral stent in comparison to catheter-associated urinary tract infections (CAUTI) and uncomplicated urinary tract infections (UTI).

**Methods:**

Google scholar, PubMed, Embase, Medline, and Cochrane literature databases were searched from inception to April 2022 to identify manuscripts on MUSC, uncomplicated UTI and CAUTI. A principal component analysis (PCA) was performed to identify patterns of the pathogen spectrum of the different groups.

**Results:**

We included 29 studies on MUSC, 28 studies on uncomplicated UTI and 23 CAUTI studies. The proportion of *Staphylococci*, *Enterococci* and *Candida* were significantly higher in MUSC and stent associated bacteriuria compared to their proportion in uncomplicated UTIs where *E. coli* dominates. By comparing MUSC, CAUTI and UTI with a PCA, the detected pathogen spectrum exhibited clearly distinguishable trends in the frequency of the main isolated pathogens influencing these three groups of urinary tract infections. With respect to MUSC and UTI, their 95% confidence interval ellipse only showed minimal overlap emphasizing that the spectrum of pathogens in the two groups is clearly distinct.

**Conclusions:**

The frequency of detection of *Staphylococci*, *Enterococci* and *Candida* is more common in MUSC as compared to UTI. Thus, patients with indwelling ureteral stents should undergo an antimicrobial prophylaxis targeting this microbial spectrum in case of further surgery.

**Supplementary Information:**

The online version contains supplementary material available at 10.1007/s00345-024-05354-x.

## Introduction

Ureteral stents are an important component of urology routine practice intended to maintain ureteral patency and to avoid obstruction of the upper urinary tract. Main indications for ureteral stent placement are urolithiasis, ureteral strictures, direct invasion or external compression by pelvic, retroperitoneal or metastatic malignancies as well as upper urinary tract carcinoma [1]. Ureteral stents are also used to prevent post-surgical complications. This makes ureteral stents indispensable devices in urology practice. However, they offer an ideal surface for microbial adhesion and biofilms are prone to develop on such materials. Indeed, antibiotic prophylaxis does not prevent stent colonization, which appears in 100% of patients with a permanent ureteral stent and in 70% of those temporarily stented [2].

In the majority of cases, microbial ureteral stent colonization (MUSC) remains asymptomatic. However, MUSC can be associated with infectious complications and is a leading risk associated with ureteral stent placement [3]. Infection associated with ureteral stents can lead to significant morbidity such as acute pyelonephritis, renal failure or urosepsis [4]. Therefore, antimicrobial prophylaxis is recommended during the placement of ureteral stents [2]. Microbial ureteral stent colonization and ureteral stent associated bacteriuria have been researched in in many studies, however the isolated pathogens differed between the different studies. Still, no consensus exists regarding the prevailing spectrum of microorganisms as well as the clinical significance of MUSC and stent associated bacteriuria. Therefore, the aim of this comprehensive review is to highlight differences in the pathogen spectrum encountered in MUSC compared to uncomplicated UTI and CAUTI. This will further allow to determine the predominant microbial spectrum in patients with an indwelling ureteral stent to optimize the choice of peri-interventional antimicrobial prophylaxis in those patients.

## Materials and methods

### Data collection

Google scholar, PubMed, Embase, Medline, and Cochrane literature databases were searched from inception to April 2022 to identify manuscripts on ureteral stent colonization. The search terms used were “(urinary tract infection, UTI or catheter-associated urinary tract infection, CAUTI) and (stent or stenting) and (pathogen or colonization)”, “infection on ureteral stent”, “ureteral stent colonization”, “ureteral stent pathogens”. A total of 6780 manuscripts were identified. Two authors performed independent scrutiny of these manuscripts and selected manuscripts to be included and selected studies were cross-checked by the same authors. Included studies had to fulfill the following criteria: (1) pathogens isolated were identified at least down to the genus level and *E. coli* down to the species level; (2) numbers of isolates or percentages of pathogens were present in the main text or supplementary material and allowed further grouping and final percentages calculation if needed; (3) material and methods section was considered clear and reproducible. A second author checked the relevance of all manuscripts. Finally, 29 studies were selected for data retrieval. We selected to retrieve the percentage of microorganisms isolated from stents and catheters and grouped them by type of microorganism. This choice was dictated by the reporting of the microbial spectrum in studies and aimed to maximize the number of studies that could be included as principal component analysis requires more samples (i.e., studies) than descriptors (i.e., pathogens included). When grouping of microorganisms or numbers of isolates were available, the percentage were calculated from the raw data.

For comparison purposes, data from studies on the search terms “uncomplicated urinary tract infection” (UTI) and “nosocomial UTI” (catheter associated urinary tract infection, CAUTI) were used for further analysis. For this purpose, Google scholar, PubMed, Embase, Medline, and Cochrane literature databases were searched from January 2002 to April 2022. We used the same inclusion criteria as described above for MUSC. In addition, we tried to select papers from different geographical origins to avoid bias due to similar populations belonging to the same locations (i.e., country). Papers with limited dataset (< 20 patients were excluded) as below such size the changes induced by 1 isolates affected the final pathogen spectrum by more than 5%.

### Statistical analysis

All data retrieved from the original publications were compiled using spreadsheet software (Libreoffice 6 or MS Excel). Basic descriptive statistics and principal component analysis (PCA) were performed using R [5]. PCA was chosen as it is a linear dimensionality reduction technique that transforms a set of correlated features in a high dimensional space (in our case the multidimensional pathogen spectrum where the percentage of each pathogen corresponds to a dimension) into a series of uncorrelated features in the low dimensional space (two dimensions in our case). The technique is useful to visualize data as reducing the dimensions of data to 2D allows us visualizing patterns contained in the datasets more clearly. Before performing the PCA care was taken to make sure that the final dateset used met the minimal requirements for PCA. Multi-normality was not met, however acceptable skewness and kurtosis were found for the data allowing to perform a PCA without biais to the analysis. Furthermore, the dataset contained more object (i.e., studies) than descriptors (i.e., pathogens) and contained an acceptable number of 0. Under those conditions, the dataset was thus considered acceptable for PCA.

Furthermore, using the raw data and the information gathered from PCA, boxplots focusing on the main identified pathogens were plotted with R. Because of non-normal distributions and inhomogeneous variances the differences between the observed percentages of those pathogens where analysed using the Kruskal–Wallis test followed by post-hoc pairwise comparison with the Wilcoxon test with Benjamini and Hochberg [6] correction for multiple testing.

## Results

We included 29 studies on MUSC from which 2201 pathogens were isolated [3, 7–34]. Similarly, for comparison purposes, we included 28 studies on uncomplicated UTI [35–60] from which 24,885 pathogens were isolated as well as 23 CAUTI [61–83] studies with 20,887 pathogens identified. Finally, 7 studies with 215 isolates from urine of patients with indwelling ureteral stents were also included [8, 9, 22, 34, 84–86]. Baseline characteristics of the included studies can be found in Table 1.

**Table 1:** Characteristics of included studies on the microbial spectrum of ureteral stent colonization (stent), catheter associated urinary tract infection (CAUTI), urinary tract infection (UTI) and urine culture on patients with indwelling ureteral stent (urine stent)

Author, year	Reference	Datapoint in Fig. 2	Pathogens isolated (n)	Type of sample (stent, urine)	Positive culture (%)	Type of microorganism detected (%)								
						*Enterococcus* spp.	*Streptococcus* spp.	*Staphylococcus* spp.	*E. coli*	*Proteus* spp.	*Enterobacteriaceae* spp.	Pseudomonas spp..	*Candida* spp.	Other
Al-Ghazo, 2010	[7]	8	29	Stent	23	0	17	0	52	7	14	0	7	3
Amine Saouli, 2021	[8]	28	38	Stent	100	18	0	13	47	0	21	0	0	0
Anak Agung, 2019	[9]	25	18	Stent	60	0	0	0	39	0	6	50	0	6
Aydin, 2016	[10]	18	30	Stent	29	3	10	40	17	0	7	0	13	10
Ben-Meir, 2009	[11]	11	82	Stent	10	33	10	26	9	0	7	10	0	12
Bonkat, 2013	[12]	5	306	Stent	8	24	1	17	9	3	4	6	14	22
Bonkat, 2013	[13]	4	200	Stent	16	17	7	19	14	2	3	1	13	24
Bonkat, 2011	[14]	1	224	Stent	23	18	8	18	12	1	4	2	10	27
Bonkat, 2012	[3]	6	26	Stent	4	31	4	19	11	0	0	4	8	23
Farsi, 1995	[15]	10	140	Stent	30	0	16	25	9	0	14	23	1	12
Garcia-Aparicio, 2015	[16]	21	43	Stent	100	12	0	5	12	14	23	21	5	7
Kehinde, 2004	[17]	2	80	Stent	7	14	0	24	29	0	0	20	13	0
Klis, 2009	[18]	7	70	Stent	26	15	0	52	7	2	4	8	1	11
Kozyrakis, 2018	[19]	19	115	Stent	74	23	6	26	17	0	7	5	0	15
Lifshitz, 1999	[20]	3	100	Stent	15	31	0	14	31	0	3	14	7	0
Matsukawa, 2005	[21]	13	37	CAUTI	30	14	3	43	16	0	13	8	8	3
Mehmet, 2021	[22]	22	67	Stent	31	27	4	15	19	0	4	9	15	6
Nevo, 2019	[23]	24	103	Stent	100	18	15	15	17	5	11	3	10	8
Paick, 2003	[24]	12	47	Stent	21	24	8	8	20	4	8	4	4	20
Rahman, 2010	[25]	14	45	Stent	21	0	2	7	36	18	21	16	0	0
Reid, 1992	[26]	15	44	Stent	27	3	3	41	3	0	3	0	0	47
Riedl, 1999	[27]	9	118	Stent	100	33	10	26	9	3	7	10	1	2
Salari, 2021	[28]	29	64	Stent	64	25	0	17	14	0	0	11	2	13
Sarier, 2017	[29]	16	24	Stent	100	58	0	13	4	0	13	0	13	3
Sarier, 2017	[30]	17	18	Stent	100	44	0	6	17	0	11	0	22	4
Shabeena, 2018	[31]	23	40	Stent	100	10	18	10	20	0	10	13	0	20
Volkan Ülker, 2019	[32]	20	8	Stent	20	23	6	26	17	0	7	5	8	7
Wang, 2021	[33]	27	39	Stent	95	15	0	5	38	0	13	10	3	15
Zhang, 2018	[34]	26	46	Stent	100	20	0	9	37	2	13	20	0	10
Ahmad, 2012	[35]	34	591	UTI	100	0	7	0	54	4	25	8	0	2
Arlene Rodriguez, 2012	[36]	31	140	UTI	100	0	0	1	76	6	12	6	0	1
Bahadin, 2011	[37]	35	333	UTI	100	2	1	4	75	3	4	2	1	8
Batra, 2020	[38]	51	295	UTI	100	2	0	4	67	2	18	4	0	3
Beyene, 2011	[39]	36	21	UTI	100	0	0	19	33	5	43	0	0	0
Bouskraoui, 2010	[40]	37	121	UTI	100	2	1	1	72	6	16	2	0	0
Cui, 2021	[41]	54	401	UTI	100	6	1	4	41	5	15	5	4	19
Dariusz Chojeta, 2021	[42]	46	285	UTI	100	5	0	0	51	14	22	3	0	5
Duicu, 2021	[43]	56	331	UTI	100	3	0	1	72	7	11	6	0	1
F.M.E. Wagenlehner, 2009	[44]	33	3018	UTI	100	3	1	9	77	5	7	0	0	2
Gordon, 2003	[45]	41	1466	UTI	100	16	0	6	43	6	20	7	0	2
Gordon, 2003	[45]	42	783	UTI	100	13	0	3	46	10	16	9	0	3
Gordon, 2003	[45]	43	531	UTI	100	4	0	3	60	7	18	6	0	2
Guclu, 2021	[46]	48	241	UTI	100	0	0	0	69	3	19	5	0	5
Huang, 2021	[47]	49	7646	UTI	100	16	4	6	43	4	9	5	0	13
Ioannou, 2020	[48]	50	205	UTI	100	13	0	3	41	12	17	5	5	4
Kolawole, 2010	[49]	45	180	UTI	100	0	0	22	31	16	8	23	0	0
Samia S. Khamees, 2012	[50]	44	256	UTI	100	0	2	11	34	22	27	3	0	1
Nath, 2021	[51]	55	49	UTI	100	12	0	16	39	4	19	4	6	0
Onifade, 2011	[52]	38	42	UTI	100	4	0	8	66	6	13	3	0	0
Oorji, 2022	[53]	53	333	UTI	100	13	5	14	50	1	15	0	0	3
Rupinder Bakshi, 2021	[54]	52	1306	UTI	100	1	0	9	49	11	26	3	0	1
Shamataj, 2012	[55]	30	411	UTI	100	0	0	10	38	1	29	3	8	11
Shams, 2017	[56]	57	152	UTI	100	1	4	13	55	0	13	0	9	6
Turnidge, 2002	[57]	32	903	UTI	100	11	2	6	38	4	21	11	0	7
Wang, 2013	[58]	39	92	UTI	100	17	2	3	64	4	6	3	0	1
Wariso, 2010	[59]	40	234	UTI	100	5	0	17	33	9	19	8	0	9
Yi-Te, 2020	[60]	47	4519	UTI	100	15	5	9	30	2	15	7	0	18
Ahmed, 2019	[61]	72	89	CAUTI	100	8	0	6	27	7	33	5	0	16
Amna Butt, 2015	[62]	80	118	CAUTI	100	0	0	7	49	10	12	14	8	0
Bi, 2009	[63]	77	450	CAUTI	100	11	0	28	39	4	10	0	0	9
Bizuaeyhu, 2022	[64]	75	88	CAUTI	100	13	0	9	6	0	6	9	45	13
Chitnis, 2012	[65]	64	5756	CAUTI	100	19	0	0	26	0	15	16	25	1
Darma-Kusuma, 2012	[66]	68	36	CAUTI	100	0	3	28	44	0	14	11	0	0
Duszynska, 2020	[67]	78	307	CAUTI	100	20	0	0	14	3	14	6	12	31
H Guanche-Garcell, 2011	[68]	60	NA	CAUTI	100	0	0	23	54	0	15	7	0	1
Joon, 2013	[69]	66	61	CAUTI	100	31	0	12	38	0	3	5	10	1
Lai, 2017	[70]	73	66	CAUTI	100	17	0	3	24	5	3	15	27	6
Lee, 2004	[71]	59	40	CAUTI	100	22	0	15	0	10	0	29	0	24
Lili Tao, 2011	[72]	61	7064	CAUTI	100	22	1	4	19	1	8	4	35	6
Milan, 2009	[73]	76	49	CAUTI	100	0	0	0	40	16	26	18	0	0
Mladenovic, 2015	[74]	62	71	CAUTI	100	13	0	1	11	4	23	20	28	0
Nirmala Poddar, 2020	[75]	74	76	CAUTI	100	22	0	4	25	11	22	8	0	8
Puri, 2002	[76]	67	73	CAUTI	100	8	0	12	33	3	15	15	14	0
Sabir, 2017	[77]	71	1070	CAUTI	100	11	0	0	52	0	23	5	0	8
Smitha Bagali, 2021	[78]	79	50	CAUTI	100	4	0	6	38	4	34	10	0	4
Taiwo, 2006	[79]	70	126	CAUTI	100	0	0	11	20	3	36	27	3	0
Talaat, 2010	[80]	58	188	CAUTI	100	13	0	2	7	2	13	7	51	5
Temitz, 2012	[81]	63	22	CAUTI	100	15	0	0	24	5	12	9	33	2
Toshie Tsuchida, 2008	[82]	69	NA	CAUTI	100	32	0	0	20	0	0	13	13	22
Wazait, 2003	[83]	65	5109	CAUTI	100	17	0	10	31	16	12	11	0	3
Altunal, 2017	[84]	84	22	Urine Stent	100	14	0	5	55	0	9	9	9	9
Amine Saouli, 2021	[8]	87	41	Urine Stent	100	17	2	15	39	0	27	0	0	0
Anak Agung, 2019	[9]	85	12	Urine Stent	37	8	0	0	25	0	0	58	0	8
He, 2021	[85]	82	21	Urine Stent	100	0	0	11	58	0	0	11	0	21
Mehmet, 2021	[22]	81	22	Urine Stent	10	23	5	5	32	0	18	14	5	4
Useok Choi, 2021	[86]	83	70	Urine Stent	100	24	4	0	30	9	13	14	0	6
Zhang, 2018	[34]	86	27	Urine Stent	100	30	0	7	33	0	11	11	4	8

In most of the MUSC studies (23 out of 29) more gram-positive microorganisms than *E. coli* were isolated from stents [3, 10–24, 26–32]. In contrast, in studies of UTIs associated with ureteral stents, E. coli accounted for 30–50% of the pathogens isolated in UTIs. [35–38, 40, 42–46, 52, 53, 56, 58] [39, 41, 45, 47–51, 54, 55, 57, 59, 60] with rather high variability between studies. Regarding studies on CAUTIs, we noticed that in a quarter of the studies (8 out of 24) gram-positive microorganisms were more present than *E. coli* [64, 67, 69, 71, 72, 74, 75, 80], while in only two studies *E. coli* accounted for more than 50% of the pathogens [68, 77].

**Fig. 1:**
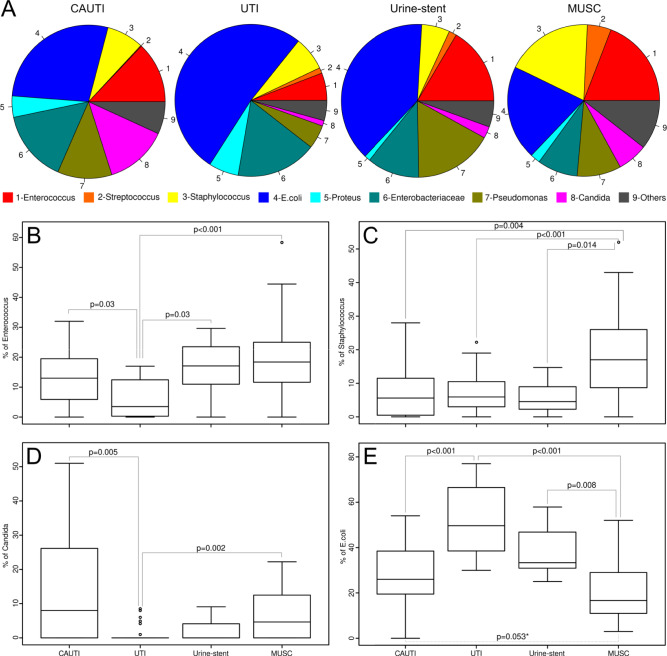
**A** Pie figure showing the spectrum of pathogens in the different groups CAUTI, UTI, urine from stent patients, MUSC. Boxplot showing the variation in the proportion of each pathogen in the different studies. **B** *Enterococcus*. **C** *Staphylococcus*. **D** *Candida*. **E** *E. coli*

Boxplots were created using the data of four pathogens (*Staphylococci* Fig. 1C, *Enterococci* Fig. 1, *Candida* Fig. 1D, E*. coli* Fig. 1E) chosen for their relevance and isolation frequency. The most prevalent pathogens isolated from ureteral stents were *Enterococci* (19%), *Staphylococci* (19%), *E. coli* (20%) and *Candida* spp. (6%). The most prevalent pathogens detected in UTI were *E. coli* (52%), *Enterococci* (6%), *Staphylococci* (7%), and *Candida* spp. (1%). The most prevalent pathogens detected in CAUTI were *E. coli* (29%), *Enterococci* (12%), *Staphylococci* (8%) and *Candida* spp. (12%). The most prevalent pathogens isolated from urine in patients with stents were *Enterococci* (16%), *Staphylococci* (6%), *E. coli* (39%) and *Candida* spp. (2%) (Fig. 1). Significant differences were detected for most of the comparisions between the different groups analyzed (Fig. 1).

**Fig. 2:**
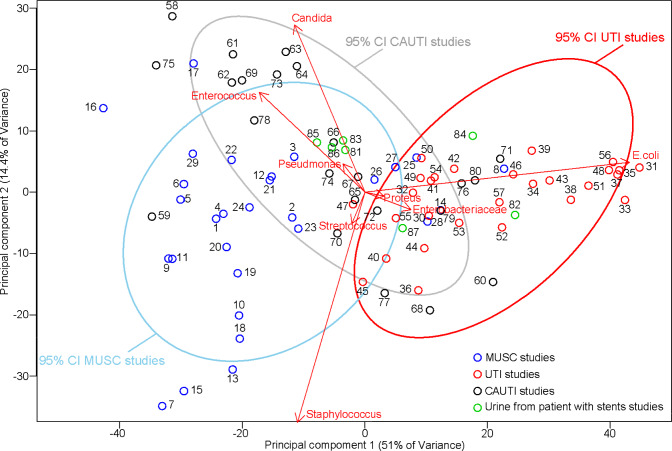
Principal component analysis showing the grouping of UTI, stent colonization and CAUTI studies. Each point represents a study with the specific pathogen spectrum described in this study (numbers correspond to the number of the study see supplementary material for details); red UTI studies, blue stent colonization studies, grey CAUTI studies. Arrows show the projections of the original features on to the principal components. 95% confidence interval ellipses were also drawn for each group. With the use of the ellipses, the grouping of the different samples in the PCA projection can be observed. Such ellipses allow understanding which data deviate from the groups formed in this PCA projection

By comparing MUSC, CAUTI data and overall UTI data using a PCA (Fig. 2), we observed that the detected pathogen spectrum exhibited clearly distinguishable trends in the frequency of the main isolated pathogens influencing these 3 groups of urinary tract infections (Fig. 2). With respect to UTIs and MUSC, their 95% confidence interval ellipse only showed minimal overlap emphasizing that the spectrum of pathogens in the two groups are clearly distinct.

Figure 2 shows that UTIs are mainly characterized by *E. coli*. All points are within the confidence interval ellipse, which means that the group is homogeneous. The isolates data from MUSC are driven towards gram-positive microorganisms with *Enterococcus* and *Staphylococcus* as major representative of bacterial infection, as well as *Candida* spp. for fungal infections.

The 95% confidence interval ellipse of CAUTIs is pointing in the direction of *Staphylococcus* and *Candida* spp. and partly intersects with the ellipse of UTIs. However, it is clearly more comparable to the ellipse of MUSC. Data from patients with ureteral stents where pathogens were collected from urine were scarce and exhibited a large variance resulting in a large 95% confidence interval ellipse not suitable for further analysis. With the current dataset, it does not seem likely that pathogens isolated are characterized by a specific spectrum. Therefore, although the data are included in the PCA, no grouping or confidence interval was drawn using those data.

## Discussion

In the present literature review, we aimed to investigate differences in pathogen spectrum detected on ureteral stents compared to patients with UTI or CAUTI. We found that different pathogen spectrums are involved in MUSC (*Staphylococci* and *Enterococci*) and possibly CAUTI compared to common UTIs where *E. coli* dominates. Consequently, bacteriuria in stented patients will likely be comprised of different pathogens than *E. coli*. Such asymptomatic bacteriuria occurring due to stent colonization is usually not considered as a risk factor, unless procedures entering the urinary tract and breaching the mucosa, particularly in endoscopic urological surgery, are considered. In addition, it needs to be considered that MUSC primarily is associated with biofilm formation on ureter stents. In many cases, these bacteria show antimicrobial resistance and MUSC cannot be identified by standard urine culture techniques as MUSC does not necessarily lead to bacteriuria. Therefore, the results shown here have potential implications for guiding peri-operative antimicrobial prophylaxis for patient with indwelling ureteral stents. In particular, antimicrobial prophylaxis prior to ureteral stent placement, ureteroscopy in patients with indwelling stents as well as change of stents in patients with long-term drainage might be more appropriate if it is also targeted against gram-positive pathogens (mostly *Staphylococci* and *Enterococci*) representing an average of 36% of isolates and up to 60% in some studies. Currently, no clear recommendations from major guidelines with respect to antimicrobial prophylaxis prior to ureteroscopy exist. This is mainly due to the low certainty of evidence as well as the lack of high-quality prospective randomized studies. Some guidelines such as the AUA 2019 guideline recommend selective antimicrobial prophylaxis based on the expected spectrum in high-risk individuals such as immunocompromised patients. Given the lack of a clear recommendations for prophylaxis in patients with indwelling ureteral stents, we suggest that a prophylaxis also covering gram-positive bacteria such as *Enterococci* and *Staphylococci* (e.g. amoxicillin-clavulanate) may be appropriate. In contrast, for patients without indwelling stents antimicrobials mainly covering the gram-negative spectrum such as first- and second-generation cephalosporins, or trimethoprim/sulfamethoxazole as a single dose are considered appropriate. Fungi represent an additional challenge in the treatment of ureteral stent associated infections. A differentiation between asymptomatic colonization, symptomatic urinary tract infection and systemic infection is relevant in deciding on the individualized treatment approach.

To investigate whether studies falling outside the 95% confidence interval of MUSC focus on a specific patient population that cannot be compared to the general population, further investigation of included patients was performed. The study no. 8 is the most extreme point that lies outside the ellipse in direction of the UTI group. The data isolated in the study represented by the study no. 8 focuses on the analysis of stent pathogens in immunosuppressed patients [7]. Also the studies no. 25 and 28 are in the ellipse of UTI, even though these studies include patients with ureteral stents. The study represented by study no. 25 were patients with diabetes mellitus and chronic kidney disease (CKD) who also had immunosuppression [9]. The study represented by study no. 28 does not deal with a particular population. Interestingly, in this study 23% of patients had CKD and 19% were diabetic [8], such conditions could lead to a shift in the pathogen spectrum.

With PCA, we observed that studies investigating pathogens in the urine of patients with indwelling stents do not follow a specific pathogen pattern [8, 9, 84]. In this context, it is important that for the identification of MUSC, an analysis of the stents seems more appropriate than urine culture. Our results suggest that urine culture is not a reliable method for identifying pathogens that colonize the stent [8, 9, 84]. These data are preliminary given the low number of datapoints that we have, but they are very much scattered.

Given that an analysis of the stent prior to change or manipulation virtually is impossible, the results of the present study provide a very helpful insight to support clinical decision making in the choice of antimicrobial prophylaxis.

The present analysis has limitations, which need to be acknowledged. Due to the exploratory nature of the study, it was not possible to fully adhere to PRISMA or other guidelines that are more fitted for univariate or bi-variate analysis in the context of network meta-analyses, meta-analyses of individual participant data, systematic reviews of harms, systematic reviews of diagnostic test accuracy studies. It is likely that this resulted in some biais. With respect to the literature retrieved, the spectrum of pathogens investigated in the included studies depends on the authors' choice and varies, especially regarding less common pathogens such as *Lactobacilli*, *Corynebacteria*, *Proteus* and others. With respect to fungi, a comparable limitation is present. Depending on the author, fungi are researched in a group or a subcategory of *Candida* species is formed. This implies that in some cases it is not possible to separate candida data from the fungi data, thus leading to exclusion of the study. As a consequence, the resulting pathogen set included in the PCA indeed present a risk of biais as it is strongly influence by the requirement of the PCA itself (not too many 0 values and more samples (i.e., studies) than descriptors (i.e., pathogens included).

In addition, the methods for the detection of MUSC may differ between sonication as well as roll-out technique which may impact the detected spectrum of microorganisms. Nevertheless, we feel that despite these limitations, our study provides helpful insight into a clinically relevant topic and may help clinical decision-making.

Future studies should include demographic data and patient data to refine the findings of the present study. Also next-generation sequencing might provide valuable help in understanding some trends observed here (in particular supporting that urine from catheterized patients provides very variable results), and provide more data on the urinary microbiota that might adhere on such catheters. Still results for next-generation sequencing can be quite long to obtain and analyse, but over time the gathered data will certainly help providing a better empirical therapy as well. Moreover, we believe that adding antimicrobial resistance data to similar studies would benefit to the overall interpretation. This will potentially help overcoming some of the limitations encountered during this review.

## Supplementary Information

Below is the link to the electronic supplementary material.

Supplementary file1 (DOCX 7 KB)

## Data Availability

All the data used for the present study are compiled in Table 1.
