# Supplementary material for: Comprehensive analysis of the bacterial spectrum for enhanced clinical insight in microbial ureteral stent colonization, uncomplicated urinary tract infections and catheter-associated urinary tract infections: a principal component analysis-based literature review
Source: World J Urol. 2024 Dec 12;43(1):29. doi: 10.1007/s00345-024-05354-x (PMC11638318; doi:10.1007/s00345-024-05354-x)
Supplement: Supplementary file 1 — Supplementary file1 (DOCX 7 KB) [file 345_2024_5354_MOESM1_ESM.docx]

**PRISMA CHECKLIST**

**Title: Identify the report as a systematic review.**

We identify our work as a PCA based literature review. All the authors believe that this is an honest description of the work provided

**INTRODUCTION**

***Rationale: Describe the rationale for the review in the context of existing knowledge.***

Microbial ureteral stent colonization and ureteral stent associated bacteriuria have been researched in many studies, however the isolated pathogens differed between the different studies. Still, no consensus exists regarding the prevailing spectrum of microorganisms as well as the clinical significance of MUSC and stent associated bacteriuria

***Objectives: Provide an explicit statement of the objective(s) or question(s) the review addresses.***

Our objective is stated at the end of the introduction: “The aim of this comprehensive review is to highlight differences in the pathogen spectrum encouneterd in MUSC compared to uncomplicated UTI and CAUTI. This will further allow to determine the predominant microbial spectrum in patients with an indwelling ureteral stent to optimize the choice of peri-interventional antimicrobial prophylaxis in those patients. “

**METHODS**

***Eligibility criteria: Specify the inclusion and exclusion criteria for the review and how studies were grouped for the syntheses.***

***Information sources: Specify all databases, registers, websites, organisations, reference lists and other sources searched or consulted to identify studies. Specify the date when each source was last searched or consulted.***

Google scholar, PubMed, Embase, Medline, and Cochrane literature databases were searched. Last date of consultation for each source was April 2022.

***Search strategy: Present the full search strategies for all databases, registers and websites, including any filters and limits used.***

The search terms used were “(urinary tract infection, UTI or catheter-associated urinary tract infection, CAUTI) and (stent or stenting) and (pathogen or colonization)”, “infection on ureteral stent”, “ureteral stent colonization”, “ureteral stent pathogens”

**Selection process: Specify the methods used to decide whether a study met the inclusion criteria of the review, including how many reviewers screened each record and each report retrieved, whether they worked independently, and if applicable, details of automation tools used in the process.**

1) Pathogens isolated were identified at least down to the genus level and E. coli down to the species level;

2) Numbers of isolates or percentages of pathogens were present in the main text or supplementary material and allowed further grouping and final percentages calculation if needed;

3) Material and methods section was considered clear and reproducible.

**Data collection process: Specify the methods used to collect data from reports, including how many reviewers collected data from each report, whether they worked independently, any processes for obtaining or confirming data from study investigators, and if applicable, details of automation tools used in the process.**

Two authors performed independent scrutiny of these manuscripts and selected manuscripts to be included and selected studies were cross-checked by the same authors. ... A second author checked the relevance of all manuscripts.

**Data items: List and define all outcomes for which data were sought. Specify whether all results that were compatible with each outcome domain in each study were sought (e.g. for all measures, time points, analyses), and if not, the methods used to decide which results to collect.**

We selected to retrieve the percentage of microorganisms isolated from stents and catheters and grouped them by type of microorganism.

Synthesis methods:

**Describe the processes used to decide which studies were eligible for each synthesis (e.g. tabulating the study intervention characteristics and comparing against the planned groups for each synthesis.**

**Describe any methods required to prepare the data for presentation or synthesis, such as handling of missing summary statistics, or data conversions.**

**Describe any methods used to tabulate or visually display results of individual studies and syntheses.**

All data retrieved from the original publications were compiled using spreadsheet software (Libreoffice 6 or MS Excel). Basic descriptive statistics and principal component analysis (PCA) were performed using R [5].

**Describe any methods used to synthesize results and provide a rationale for the choice(s). If meta-analysis was performed, describe the model(s), method(s) to identify the presence and extent of statistical heterogeneity, and software package(s) used.**

Basic descriptive statistics and principal component analysis (PCA) were performed using R [5]. PCA was chosen as it is a linear dimensionality reduction technique that transforms a set of correlated features in a high dimensional space (in our case the multidimensional pathogen spectrum where the percentage of each pathogen corresponds to a dimension) into a series of uncorrelated features in the low dimensional space (2 dimensions in our case).

**Describe any methods used to explore possible causes of heterogeneity among study results (e.g. subgroup analysis, meta-regression).**

Studies outsied the 95CI ellipses were investigated separately. It appeared that immunosuppression could be an important factor as stated in the discussion

**RESULTS**

**Study selection: Describe the results of the search and selection process, from the number of records identified in the search to the number of studies included in the review, ideally using a flow diagram.**

We included 29 studies on MUSC from which 2201 pathogens were isolated [3, 7-34]. Similarly, for comparison purposes, we included 28 studies on uncomplicated UTI [35-60] from which 24885 pathogens were isolated as well as 23 CAUTI [61-83] studies with 20887 pathogens identified. Finally, 7 studies with 215 isolates from urine of patients with indwelling ureteral stents were also included [8, 9, 22, 34, 84-86].

NO FLOWCHART DONE

**Cite studies that might appear to meet the inclusion criteria, but which were excluded, and explain why they were excluded.**

We included all studies that fitted inclusion criteria

**Study characteristics :Cite each included study and present its characteristics.**

All studies are properly cited and references are provided.

Also extracted data are provided as supplementary material

**DISCUSSION**

**Provide a general interpretation of the results in the context of other evidence.**

DONE

**Discuss any limitations of the evidence included in the review.**

DONE

**Discuss any limitations of the review processes used.**

DONE

**Discuss implications of the results for practice, policy, and future research.**

DONE

**NOT DONE OR NOT RELEVANT FOR THIS WORK**

**Describe any sensitivity analyses conducted to assess robustness of the synthesized results.**

**Reporting bias assessment**

**Describe any methods used to assess risk of bias due to missing results in a synthesis (arising from reporting biases).**

**Describe any methods used to assess certainty (or confidence) in the body of evidence for an outcome.**

**Specify the methods used to assess risk of bias in the included studies, including details of the tool(s) used, how many reviewers assessed each study and whether they worked independently, and if applicable, details of automation tools used in the process.**

**Specify for each outcome the effect measure(s) (e.g. risk ratio, mean difference) used in the synthesis or presentation of results.**
